# *In situ* study on interactions between hydroxyl groups in kaolinite and re-adsorption water†

**DOI:** 10.1039/d0ra01905d

**Published:** 2020-04-30

**Authors:** Yanna Han, Zhuangzhuang Yan, Lijun Jin, Junjie Liao, Guorui Feng

**Affiliations:** College of Mining Engineering, Taiyuan University of Technology Taiyuan 030024 China fguorui@163.com +86 351 6010177; State Key Laboratory Breeding Base of Coal Science and Technology Co-founded by Shanxi Province and the Ministry of Science and Technology, Taiyuan University of Technology Taiyuan 030024 China liaojjie@163.com +86 351 6010482; State Key Laboratory of Fine Chemicals, Institute of Coal Chemical Engineering, School of Chemical Engineering, Dalian University of Technology Dalian 116024 China

## Abstract

The interactions between O–H groups in kaolinite and re-adsorption water is an important aspect that should be considered in the hydraulic fracturing method for the production of shale gas, because the external water adsorbed by kaolinite in shale would significantly affect the desorption of methane. In this study, the interactions were investigated *via* changing the amount of O–H groups and re-adsorption water in kaolinite by heating treatment and water re-adsorption. To overcome the overlap of IR vibration bands of the O–H functional groups in H_2_O and those in parent kaolinite, kaolinite samples with D_2_O re-adsorption were prepared by drying the H_2_O from raw kaolinite and soaking the dried kaolinite in D_2_O. The interactions between O–H groups in kaolinite and D_2_O molecules were investigated by *in situ* DRIFT and TG-MS. The results demonstrated that the vibration at 3670 ± 4 cm^−1^ in the DRIFT spectra could be due to the outer O–H groups of the octahedral sheet on the upper surface of the kaolinite microcrystal structure, rather than a type of inner-surface O–H group. All types of O–H groups, including the inner O–H groups in kaolinite, could be transformed into O–D groups after D_2_O re-adsorption at room temperature. The inner-surface O–H groups in kaolinite are the most preferred sites for D_2_O re-adsorption; thus, they would be the key factor for studying the effect of re-adsorption water on methane desorption. When the temperature increased from 100 °C to 300 °C, two layers of kaolinite slipped away from each other, resulting in the transformation of inner-surface O–H groups into outer O–H groups. Thus, the temperature range of 100 to 300 °C was suggested for the heat treatment of kaolinite to decrease the content of inner-surface O–H groups; thereby, the amount of re-adsorption water was reduced. However, to thoroughly remove the re-adsorption water, a temperature higher than 650 °C should be used.

## Abstract 2

Because two layers slipped away from each other, inner-surface O–H was transformed into outer O–H during heating from 100–300 °C. Re-adsorption water could be thoroughly removed at 650 °C.

## Graphical abstract

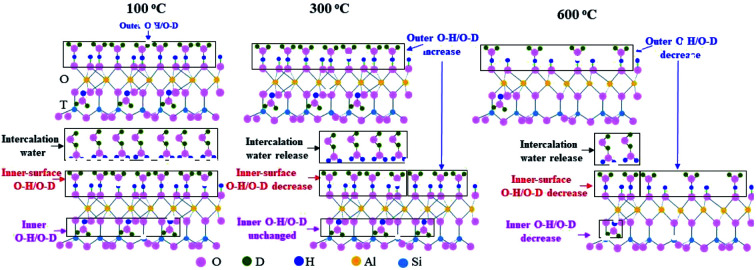

## 1 Introduction

Water obviously affects the desorption behaviors of shale gases from clay minerals.^1–4^ The hydraulic fracturing method could take external water into shale.^5,6^ Thus, it would be of practical meaning to study the effect of the interaction between re-adsorption water and shale on shale gas behaviors. Kaolinite, as one of main components in clay minerals, interacts strongly with water through hydrogen bonds.^3,7–9^ Ledoux *et al.*^10^ summarized the four types of O–H groups in kaolinite, which include two types of outer O–H groups, located on the broken-edge site and on the upper surface of the kaolinite microcrystal structure; the inner-surface O–H groups, which exist on the surface of the octahedral sheets opposite the tetrahedral oxygen; and the inner O–H groups. Different O–H groups have different energies of bonding with water. The water behavior could be changed by changing the O–H groups. Therefore, a study on the interactions between O–H groups in kaolinite and water would provide fundamental understanding to control the interaction between water and kaolinite in shale, and thus to enhance the amount of methane desorbed from clays.^3,4,11^

Previous studies were carried out using D_2_O to investigate the assignment of the O–H groups in kaolinite.^10,12–15^ However, scant reports use D_2_O instead of water to study the re-adsorption water behavior in kaolinite. Diffuse reflectance infrared Fourier transform (DRIFT) has been widely used to observe the O–H and O–D group vibrations in kaolinite^16,17^ and lignite.^18,19^ In our previous studies,^18,19^*in situ* DRIFT and thermogravimetry coupled with mass spectrometry (TG-MS) were employed to research the heating process of lignite containing re-adsorption D_2_O. The interactions between hydrogen bonds in lignite and re-adsorption water were investigated in detail. In this study, the two instruments were also applied to research the heating process of kaolinite containing re-adsorption D_2_O. The changes in DRIFT spectra of O–H and O–D groups and the release of ion-related D during the heating process of kaolinite was systematically analyzed.

The exchange of hydrogen by deuterium results in a lower wavenumber of O–D bonds corresponding to the O–H groups.^12,13^ Ledoux *et al.*^10^ revealed that after kaolinite was heated at 200 °C and deuterated under vacuum, the intensity of three O–H peaks at 3695 cm^−1^, 3670 cm^−1^, and 3650 cm^−1^ decreased significantly, whereas the 3620 cm^−1^ peak was not obviously affected. They assigned the former three peaks as inner-surface groups and the last peak as an inner O–H group. Mlchaelian *et al.*^14^ reported that inner-surface O–H groups are more readily exchanged, so that after deuteration, the peaks at 3651 cm^−1^, 3669 cm^−1^, and 3695 cm^−1^ easily shifted to 2691 cm^−1^, 2706 cm^−1^, and 2725 cm^−1^, respectively. However, according to the investigations of De Donato^13^ and Rouxhet,^15^ the inner O–H group was also exchanged with O–D after natural and low deuteration in kaolinite at room temperature. Therefore, the exchange behaviors of inner O–H groups with O–D groups at room temperature were investigated in this paper. The 3697 cm^−1^ peak could be assigned to the inner-surface O–H groups oriented perpendicular to the octahedral sheet.^10,20^ However, there are some conflicts on the assignment of O–H groups at 3669 cm^−1^ and 3652 cm^−1^. The two peaks were attributed to two inner-surface O–H groups approximately parallel to the octahedral sheet, according to the study of Kloprogge.^20^ Farmer *et al.*^21^ assigned the two peaks as the in-plane vibrations of the inner-surface O–H groups. However, Johansson *et al.*^22^ reported that the peak at 3674 cm^−1^ could represent outer-surface O–H groups on the surface of the kaolinite. In this paper, the properties of the O–H groups at 3669 cm^−1^ and 3652 cm^−1^ were studied.

In this study, D_2_O instead of H_2_O was used to soak dried kaolinite. The DRIFT spectra of O–H and O–D bands and the release of ions of D, HD, OD/H_2_O and HOD during the heating of kaolinite containing re-adsorption D_2_O were detected by *in situ* DRIFT and TG-MS, respectively. Kaolinites heated at different temperatures were characterized by X-ray diffraction (XRD) and X-ray fluorescence (XRF) to study the changes in crystal structure and elemental content of kaolinite during the heating process.

## 2 Experimental

### 2.1 Preparation of samples

Raw kaolinite was purchased from Sigma-Aldrich (Irvine, UK). Its particle size was smaller than 74 μm. As samples need to be dried before XRF testing, the XRF analysis of dried kaolinite instead of that of raw kaolinite was analyzed, as shown in Table 1. As can be seen, the minor ionic impurities in the kaolinite were Fe, Ti, Ca, Na and K. The content of SO_3_ indicated that alunite could exist in the raw kaolinite.^23^

**Table 1:** XRF analysis of dried kaolinite (wt%)

SiO_2_	Al_2_O_3_	Fe_2_O_3_	TiO_2_	K_2_O	SO_3_	Na_2_O	CaO	Others
49.03	48.41	0.47	0.22	0.47	0.76	0.15	0.06	0.43

In order to minimize the interference of H_2_O on the DRIFT spectra of kaolinite, the raw kaolinite sample was initially dried at 110 °C to remove H_2_O. The dried kaolinite was soaked in D_2_O (99.9% D, Aladdin Reagent) to prepare the kaolinite sample containing re-adsorption D_2_O. The soaking was carried out at room temperature for 7 days to ensure the full exchange of O–H groups with O–D groups. The samples of dried kaolinite and kaolinite containing re-adsorption D_2_O were denoted DK and K-D_2_O, respectively.

In order to analyze the changes in crystal structure and elemental content during the dehydroxylation of kaolinite, the DK sample was heated at the different temperatures of 500 °C, 600 °C and 650 °C to prepare the DK-500, DK-600 and DK-650 samples, respectively, for XRD and XRF analyses.

### 2.2 Analyses of the interactions between hydroxyl groups in kaolinite and D_2_O

The interactions between hydroxyl groups in kaolinite and D_2_O were investigated using *in situ* DRIFT obtained using the Vertex 70 infrared spectrometer (Bruker Co. Ltd., Germany) and TG-MS apparatus (Setsys Evolution, SETARAM, France).

The DRIFT spectra of DK and K-D_2_O employed at 30 °C and the *in situ* DRIFT spectra measured at 100 °C, 200 °C, 300 °C, 400 °C, 500 °C, 600 °C and 650 °C of K-D_2_O during heating were recorded in the wavenumber range of 600–4000 cm^−1^ with the resolution of 4 cm^−1^. The spectra of DRIFT and *in situ* DRIFT were measured with a Vertex 70 infrared spectrometer (Bruker Co. Ltd., Germany), and the reaction cell was the 0030-102 high-pressure/high-temperature accessory (Pike Co. Ltd., USA) with ZnSe windows. The detailed parameters for the spectrometer and the measurement process can be found in our previous studies.^18,19^ Specifically, DK and K-D_2_O were fully ground for 30 min under N_2_ protection before each run. About 30 mg of the ground DK and 40 mg of ground K-D_2_O were used for the tests of DRIFT and the *in situ* DRIFT, respectively. The spectra were recorded over the range of 600–4000 cm^−1^ at a resolution of 4 cm^−1^.

In order to verify the findings from *in situ* DRIFT, the D, HD, H_2_O/OD and HOD released during the heating of K-D_2_O were detected by the TG-MS apparatus. For each run, 20.0 ± 0.5 mg K-D_2_O sample was heated from 30 °C to 700 °C with the heating rate of 10 °C min^−1^ using 100 mL min^−1^ high-purity He as the carrier gas. The test was repeated at least twice, and the detailed procedure can be found in the previous study.^8^ During the TG-MS analysis, the TG and DTG curves could be obtained, and the D-containing substances released were simultaneously analyzed by MS.

### 2.3 Characterization of samples

To study the crystal structure change of kaolinite during dehydroxylation, the DK, DK-500, DK-600 and DK-650 samples were characterized using XRD equipment (Bruker D2 Phaser desktop X-ray diffractometer). The diffraction conditions were Cu Kα_1_/α_2_ radiation, a 30 kV tube voltage and 10 mA tube current.

The chemical compositions of DK, DK-500, DK-600 and DK-650 were analyzed by XRF (Bruker S8 TIGER) according to the method provided by ASTM D6349.^24^

The pore structure of DK was measured by N_2_ adsorption at −196 °C (ASAP 2020, Micromeritics, USA). The calculation of specific surface area (*S*_BET_) was done according to Brunauer Emmett Teller (BET) equation. The volume of total pores (*V*_total_) and macropores (*V*_macro_) were calculated using the BJH equation according to the adsorption curve.

## 3 Results and discussion

### 3.1 Physical and chemical properties of kaolinite

As shown in Table 2, the *S*_BET_ of kaolinite is 18.2 m^2^ g^−1^, which is between that of low-defect kaolinite (11.7 m^2^ g^−1^ (ref. 25) or 16.1 m^2^ g^−1^)^26^ and that of high-defect kaolinite (23.5 m^2^ g^−1^, 23.1 m^2^ g^−1^ or 22.4 m^2^ g^−1^)^26^, indicating that the sample chosen in this study possesses both the properties of low- and high-defect kaolinite. The values of *V*_total_ and *V*_macro_ were 10.4 cm^3^ g^−1^ and 6.2 cm^3^ g^−1^ respectively, suggesting that pores in the kaolinite mainly existed as macropores. Water in kaolinite could exist in different forms. Water molecules could be confined in microplatelets or in contact with the central platelets of kaolinite.^27^ Water in macropores is bulk (free) water, which possesses almost the same properties as pure water.^28^ Thus, a high proportion of macropores in the kaolinite could imply that the impact of pores on the properties of re-adsorption water is lower than that of kaolinite possessing high micropore content.

**Table 2:** Pore structures in kaolinite

*S*_BET_ (m^2^ g^−1^)	*V*_total_ × 10^−3^ (cm^3^ g^−1^)	*V*_macro_ × 10^−3^ (cm^3^ g^−1^)
18.2	10.4	6.2

**Fig. 1:**
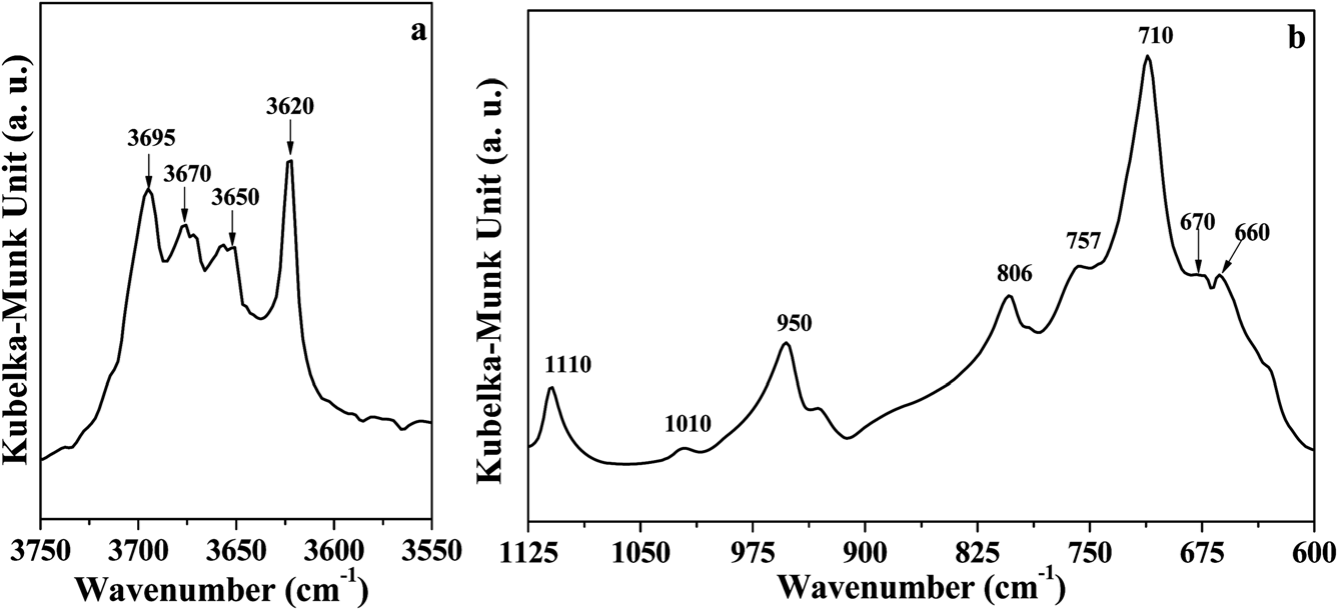
DRIFT spectra of dried kaolinite (a) O–H region; (b) Si–O and Al–O region.

The DRIFT spectrum of DK is displayed in Fig. 1. The narrow band around 3620 cm^−1^ (Fig. 1a) represents the inner O–H groups' vibration in kaolinite.^5^ The peak at 3695 cm^−1^ is assigned to the inner-surface O–H groups oriented perpendicular to the octahedral sheet.^10,13,21^ For the peaks at 3670 cm^−1^ and 3650 cm^−1^, Farmer *et al.*^21^ assigned the two peaks to the in-plane vibrations of inner-surface O–H groups, whereas Johansson *et al.*^22^ reported the peaks at 3674 cm^−1^ could represent outer surface O–H groups on the surface of the kaolinite. Ledoux *et al.*^10^ pointed out two types of outer O–H groups in kaolinite. The two types of outer groups were located on the broken edge site and on the upper surface of kaolinite microcrystal structure. In this study, the peak at 3670 cm^−1^ is assigned to the outer surface O–H groups on the surface of kaolinite microcrystal structure, and the peak at 3650 cm^−1^ could be the other type of outer O–H groups. The reason for the assignment of the two peaks will be discussed below.

As displayed in Fig. 1b, the peak at 1110 cm^−1^ was assigned to the stretching mode of apical Si–O, and the band at 1010 cm^−1^ was caused by the stretching vibrations of Si–O–Si.^29,30^ Several adsorption peaks appeared at lower wavenumbers: 950 cm^−1^ (surface Al–OH–Al bonds),^17,31^ 806 cm^−1^ (amorphous silica),^30^ 757 cm^−1^ (Si–O–Al),^30^ 710 cm^−1^ (O–Si–O),^29^ and 660–670 cm^−1^ (symmetric Al–O).^32,33^

### 3.2 Interaction between re-adsorption water and hydroxyl groups of kaolinite

#### 3.2.1 Effect of re-adsorbed water on hydrogen bonds in kaolinite

**Fig. 2:**
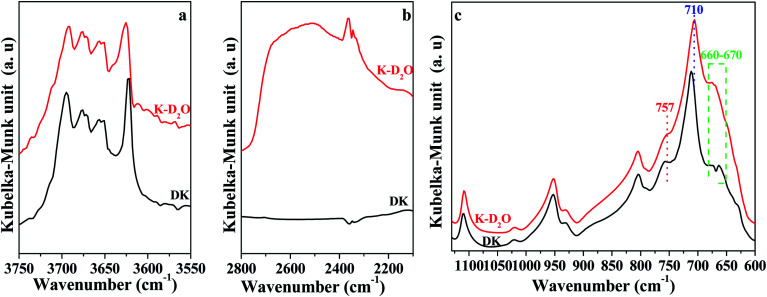
DFTIR spectra of DK and K-D_2_O at 30 °C. (a) O–H region; (b) O–D region; (c) Si–O and Al–O region.

The DRIFT spectra of DK and K-D_2_O at 30 °C are shown in Fig. 2. The significant difference between the spectra of DK and K-D_2_O samples indicate that the water re-adsorption behaviors of kaolinite could be revealed through the isotopic substitution method. Compared to DK, there was a broad prominent band centered at approximately 2500 ± 10 cm^−1^, which could be assigned to O–D vibration^18,34^ (Fig. 2b). The intensities of peaks at 3695 cm^−1^ (inner-surface O–H group) and at 3620 cm^−1^ (inner O–H group) were weak for K-D_2_O (Fig. 2a). The results indicate that there were interactions between re-adsorption D_2_O and O–H groups on the inner-surface sites and the inner sites of kaolinite. As shown in Fig. 2c, the peaks at 757 cm^−1^ (Si–O–Al) and 710 cm^−1^ (O–Si–O) shifted to lower wavenumbers, whereas the peaks at 660–670 cm^−1^ (symmetric Al–O) shifted to higher wavenumbers. According to the kaolinite structure, the interaction between re-adsorption D_2_O and inner O–H groups could affect the lower wavenumber transformation of Si–O–Al and O–Si–O, because the two bonds exist in the internal structure of kaolinite. The higher wavenumber transformation of symmetric Al–O could be due to the interaction between re-adsorption D_2_O and inner-surface O–H groups, as the symmetric Al–O could directly connect with inner-surface O–H groups. The results indicated that the three kinds of bonds were affected when soaking the dried kaolinite in D_2_O at room temperature.

#### 3.2.2 Effect of re-adsorbed water on hydrogen bonds in kaolinite during heating

**Fig. 3:**
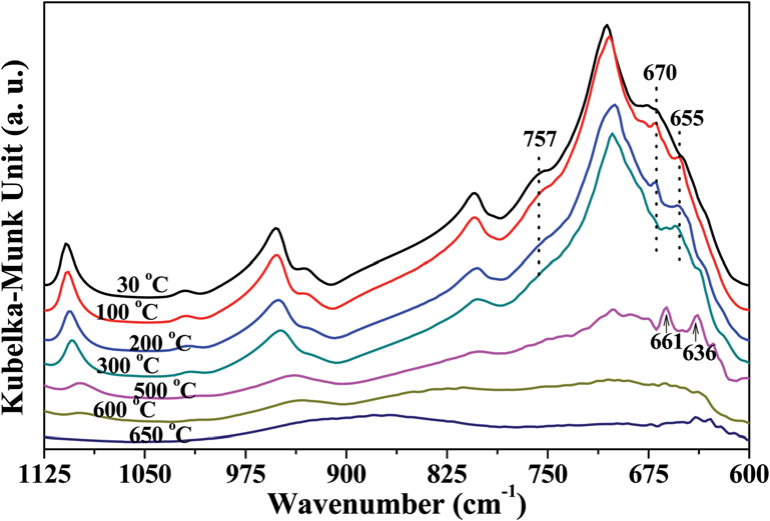
*In situ* DRIFT spectra of Si–O and Al–O region of K-D_2_O.

The changes in O–H groups and re-adsorption D_2_O of kaolinite were revealed according to the *in situ* DRIFT spectra of K-D_2_O. The variation in skeleton structure of kaolinite could be useful to revealing the reason for the changes in O–H groups and re-adsorption D_2_O, so the *in situ* DRIFT spectra of Si–O and Al–O region were also analyzed in detail, as shown in Fig. 3.

Fig. 3 shows the peaks around 757 cm^−1^ and 670 cm^−1^ related to Si–O–Al and Al–O, respectively, which gradually decreased as heating temperature increased from 30 °C to 300 °C, indicating that the kaolinite skeleton structure could be slightly affected at this stage. When the temperature was higher than 500 °C, the intensity of the peaks around 1110 cm^−1^, 1010 cm^−1^, 950 cm^−1^, 806 cm^−1^ and 710 cm^−1^ significantly declined. This indicated that the crystal structure of kaolinite initially collapsed at 500 °C. At 500 °C, the peak around 655 cm^−1^ (Si–O–Si)^23,24^ shifted to 661 cm^−1^, and a new peak at 636 cm^−1^ arising from [AlO_4/2_]^−^ (ref. 35) was found. It is reported that the first decomposition stage of kaolinite occurs at around 500 °C, during which water is eliminated and the octahedral sheet collapses.^36^ This could be the reason for the appearance of a new peak arising from [AlO_4/2_]^−^. When the temperature was raised to 650 °C, the bonds relating to Si and Al almost disappeared, indicating that heating to 650 °C could obviously destroy the crystal structure of kaolinite.

**Fig. 4:**
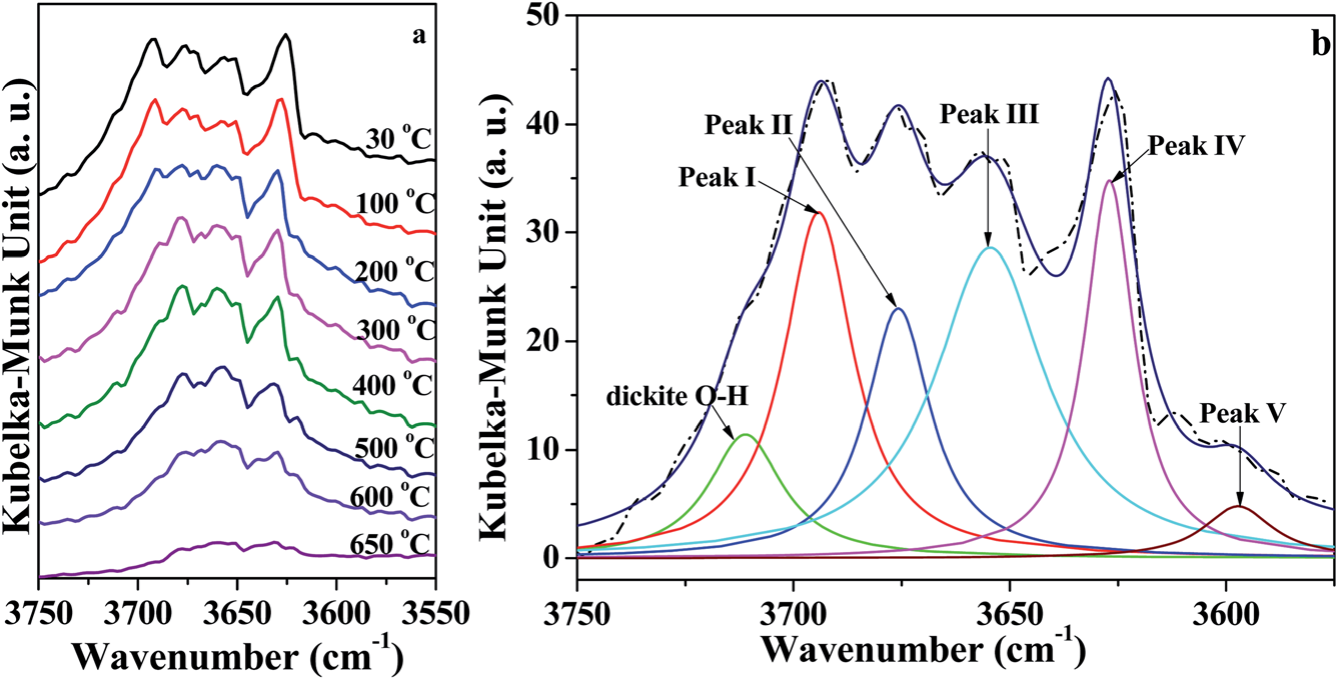
(a) *In situ* DRIFT spectra of O–H region of K-D_2_O and (b) deconvolution of O–H region obtained at 30 °C.

As illustrated in Fig. 4a, the intensity of peaks in the range of 3750–3550 cm^−1^ corresponding to O–H vibration roughly decreased with increasing temperature. In order to quantitatively analyze the changes of the O–H groups, the region of O–H (3750 cm^−1^ to 3550 cm^−1^) vibration in *in situ* DRIFT spectra was deconvoluted into six Lorentzian peaks (Fig. 4b and S1†), because Lorentzian shape was reported very suitable for the deconvolution of DRIFT spectra of O–H groups in kaolinite.^37^ For the bands, five of them are due to O–H vibration, designated as follows: peak I around 3695 ± 2 cm^−1^ (inner-surface O–H groups), peak II around 3670 ± 4 cm^−1^, peak III around 3652 ± 3 cm^−1^, peak IV around 3620 ± 5 cm^−1^ (inner O–H group), and peak V around 3580 ± 5 cm^−1^ (O–H group in intercalated water).^17^ The band at 3720 ± 5 cm^−1^ was probably caused by the presence of trace amounts of dickite.^37^ Moreover, for the deconvolution, the initial half-widths for these bands were used according to the references^7,31^ and our previous studies.^18,38,39^ Peak areas of different O–H groups in the *in situ* DRIFT spectra obtained at different temperatures for the K-D_2_O sample are summarized in Table 3. When the heating temperature increased from 30 °C to 100 °C, the areas of the five peaks remained almost unchanged, indicating that the O–H groups in kaolinite structure were intact at this stage. The peak V area significantly decreased at 300 °C, suggesting that the intercalated water was removed.

**Table 3:** Peak area of different O–H groups in *in situ* DRIFT spectra obtained at different temperatures of K-D_2_O sample

Temperature (°C)	Peak area				
	Peak I	Peak II	Peak III	Peak IV	Peak V
30	903 ± 10	646 ± 8	1011 ± 13	606 ± 10	339 ± 10
100	897 ± 8	686 ± 6	1077 ± 15	605 ± 8	337 ± 10
200	708 ± 7	714 ± 7	1059 ± 15	598 ± 7	329 ± 10
300	353 ± 8	707 ± 6	1021 ± 13	585 ± 8	154 ± 5
400	256 ± 7	690 ± 8	1009 ± 10	582 ± 5	94 ± 7
500	189 ± 7	482 ± 3	877 ± 10	574 ± 6	80 ± 6
600	136 ± 6	478 ± 5	826 ± 8	452 ± 5	45 ± 7
650	4 ± 3	159 ± 5	329 ± 5	206 ± 5	23 ± 4

**Fig. 5:**
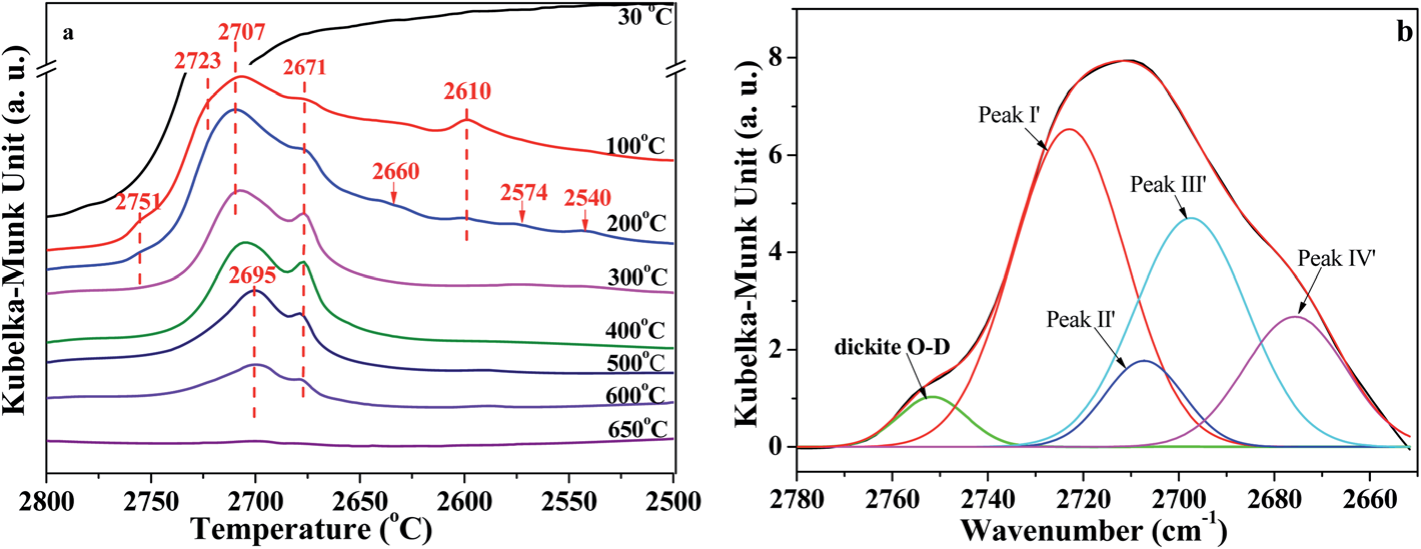
(a) *In situ* DRIFT spectra of O–D region of K-D_2_O and (b) deconvolution of O–D region obtained at 100 °C.

*In situ* DRIFT spectra of the O–D region are shown in Fig. 5. The intensity also declined with the increase of temperature. As can be seen from Fig. 5a, the bands around 2723 cm^−1^, 2707 cm^−1^, 2695 cm^−1^, 2671 cm^−1^, and 2610 cm^−1^ can be assigned to the vibrations of O–D groups. These O–D bonds have a one-to-one correspondence relationship with the bands of O–H vibration at 3695 cm^−1^, 3670 cm^−1^, 3650 cm^−1^, 3620 cm^−1^ and 3580 cm^−1^, respectively, which could be proved by the isotopic ratio of *ν*_(OH)_/*ν*_(OD)_ reported from 1.352 to 1.374.^12,18,40^ Also, based on the isotopic ratio of 3720/2751 = 1.352, the O–D bands' vibration at 2751 cm^−1^ could be attributed to the D_2_O adsorbed on dickite. The bonds at 2660 cm^−1^, 2574 cm^−1^ and 2540 cm^−1^ could be because of the imperfections in the kaolinite structure that were occupied by re-adsorption D_2_O.^40^ As shown in Fig. 5a, the intensities of bonds at 2610 cm^−1^ disappeared at 300 °C, indicating that the intercalated re-adsorption D_2_O could be removed at this stage. The peaks at 2751 cm^−1^, 2660 cm^−1^, 2574 cm^−1^ and 2540 cm^−1^ disappeared at 300 °C, indicating that O–D groups bonded at these sites could also be removed. The areas of O–D vibration at the region of 2800 cm^−1^ and 2650 cm^−1^ have almost disappeared at 650 °C, suggesting the majority of adsorption D_2_O could be removed at this temperature.

Similar to the analysis of O–H region, the O–D region (2800 cm^−1^ to 2650 cm^−1^) was also deconvoluted in the same way, as shown in Fig. 5b and S2 in ESI.† There were four peaks in Fig. 5b, peak I′ at 2723 ± 3 cm^−1^, peak II′ at 2707 ± 3 cm^−1^, peak III′ at 2695 ± 3 cm^−1^, and peak IV′ at 2671 ± 2 cm^−1^. The results demonstrated that all the four types of O–H groups, including the inner O–H groups, were exchanged with O–D groups during the D_2_O re-adsorption process of kaolinite. Peak areas of different O–D groups in *in situ* DRIFT spectra obtained at different temperatures of K-D_2_O sample are summarized in Table 4. The O–D groups for K-D_2_O heated at 30 °C were not fitted because there was a large amount of free D_2_O in K-D_2_O; thus, these groups cannot be identified very well at the moment.

**Table 4:** Peak area of different O–D groups in *in situ* DRIFT spectra obtained at different temperatures of K-D_2_O sample

Temperature (°C)	Peak area			
	Peak I′	Peak II′	Peak III′	Peak IV′
100	193 ± 10	37 ± 6	138 ± 10	58 ± 10
200	137 ± 3	92 ± 4	128 ± 8	56 ± 8
300	63 ± 4	68 ± 3	112 ± 10	45 ± 9
400	51 ± 3	55 ± 4	106 ± 5	43 ± 4
500	44 ± 2	46 ± 2	62 ± 5	44 ± 8
600	21 ± 1	23 ± 1	32 ± 3	24 ± 2
650	0.3 ± 0.2	2 ± 0.3	3 ± 0.5	1 ± 0.5

**Fig. 6:**
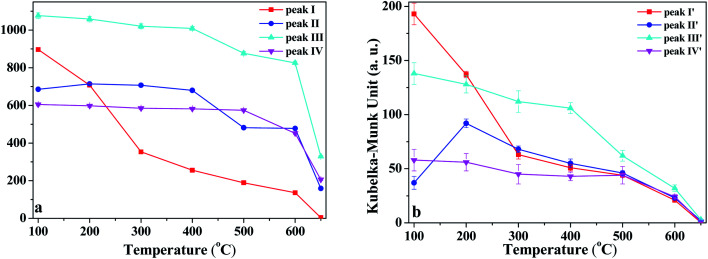
Peak area change of (a) O–H and (b) O–D groups during heating of K-D_2_O.

In order to discuss the changes in the O–H and O–D groups during the heating of K-D_2_O, the changes in peak area for the four O–H groups and four O–D groups are shown in Fig. 6. At 100 °C, the area sequence for the different O–H groups in Fig. 6a is as follows: O–H groups corresponding to peak III > inner-surface O–H groups (peak I) > O–H groups corresponding to peak II > inner O–H groups (peak IV), whereas the area sequence for different O–D groups in Fig. 6b followed the inner-surface O–D groups (peak I′) > O–D groups corresponding to peak III > inner O–D groups (peak IV′) > O–D groups corresponding to peak II′. The results indicated that the ability to re-adsorb water was different for different O–H groups, and the majority of re-adsorption D_2_O was initially adsorbed on the inner-surface sites of kaolinite. At 650 °C, the inner-surface O–H groups (peak I) approximately disappeared, whereas the other three O–H groups still existed, indicating that the properties of O–H groups corresponding to peak II and III, and those of inner O–H groups (peak IV), were different from the properties of inner-surface O–H groups. Peak II was reported to be a type of outer O–H group^10,22^ or a type of inner-surface O–H group.^20,21^ Thus, it was concluded that peak II could be assigned as an outer O–H group rather than an inner-surface O–H group.

As temperature increased from 100 °C to 300 °C, the area of inner-surface O–H groups (peak I) and that of inner-surface O–D groups (peak I′) significantly decreased, while the areas of peaks III and III′ and peaks IV and IV′ remained almost unchanged considering fitting errors, whereas the peak II area slightly increased and the peak II′ area at 200 °C and 300 °C became significantly higher than that at 100 °C. The area decrease of peaks I and I′ and area increase of peaks II and II′ indicated that peak I shifted to peak II, and peak I′ correspondingly changed into peak II′. Kristóf and Redaoui^41,42^ reported that proton delocalization and predehydroxylation of kaolinite could occur between the temperatures of 30 °C to 360 °C. The results of Fig. 3 also proved that the kaolinite skeleton structure could be slightly affected as heating temperature increased from 30 °C to 300 °C, based on the changes of peak intensity at 757 cm^−1^ (Si–O–Al) and 670 cm^−1^ (Al–O). Thus, the transformation between peak I/I′ and peak II/II′ could be explained as the transformation of O–H/O–D groups on the inner-surface (peak I/I′) into outer O–H/O–D groups of the octahedral sheet found on upper surface of the microcrystal structure (peak II/II′). The transformation could be due to the two layers of kaolinite slipping away from each other.

From 300 °C to 650 °C, the areas of peaks I and I′ continuously decreased, whereas the areas of peaks II, III and IV approximately kept unchanged until 400 °C. At 500 °C, the areas of peaks II and III dramatically decreased, indicating the initial collapse of kaolinite crystal structure and the start of dehydroxylation. The areas of peaks II′ and III′ were also decreased at 500 °C, suggesting that the outer O–D groups on the upper surface of kaolinite microcrystal structure and the O–D group's vibration at peak III′ were also removed at the initial dehydroxylation stage. The areas of peak IV and IV′ decreased at 600 °C, suggesting that the inner O–H and O–D groups can be obviously removed during the dehydroxylation. At 650 °C, the four O–D bonds almost disappeared, indicating that the re-adsorption D_2_O was approximately thoroughly removed at this temperature. The trace content of O–D bonds could be because of the inhomogeneous dehydroxylation.^43^

### 3.3 Thermal decomposition behaviors of kaolinite

**Fig. 7:**
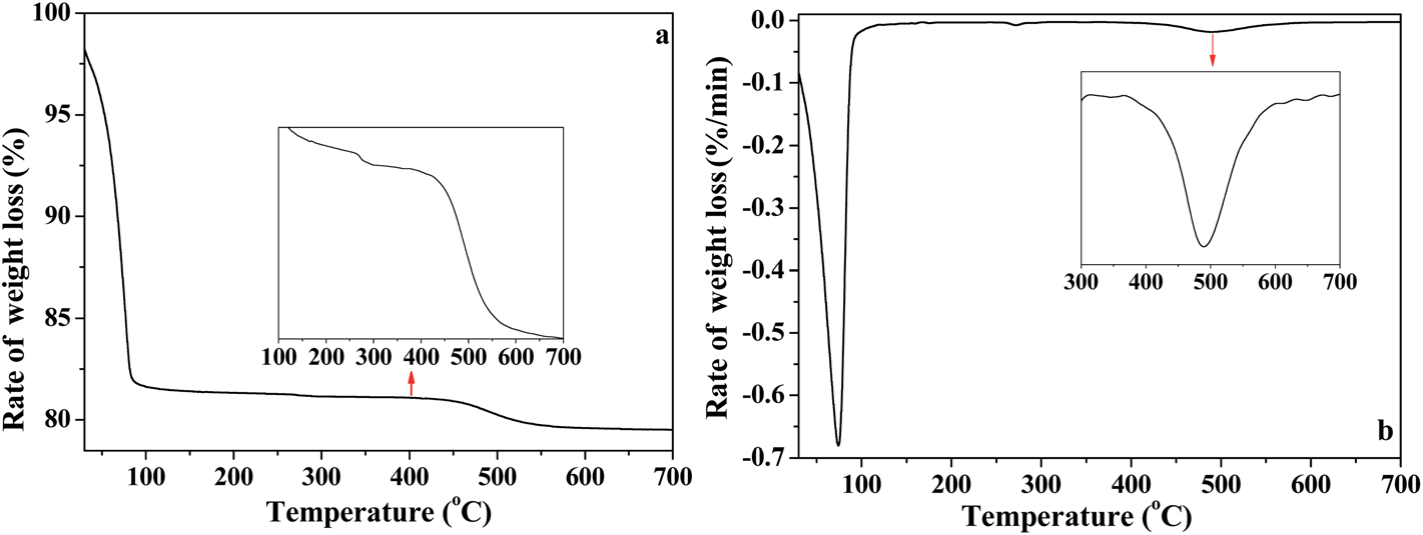
(a) TG and (b) DTG curves of K-D_2_O.

In order to further analyze the interaction of water with kaolinite, the D, HD, H_2_O/OD and HOD released during the heating process of K-D_2_O from 30 °C to 700 °C were measured by TG-MS. As shown in Fig. 7a, there were three weight loss steps in the TG curve. The first weight loss appearing at 75 °C (Fig. 7b) was due to the evaporation of free adsorption D_2_O. The second weight loss happened from 150 °C to 450 °C, which could be assigned to the predehydration process, suggesting reorganization in the octahedral layer, because reorganization was reported to always accompany the release of the intercalated water in kaolinite.^44,45^ The third step of weight loss, at 450 °C to 650 °C, was attributed to the dehydroxylation of kaolinite and the formation of metakaolinite.

**Fig. 8:**
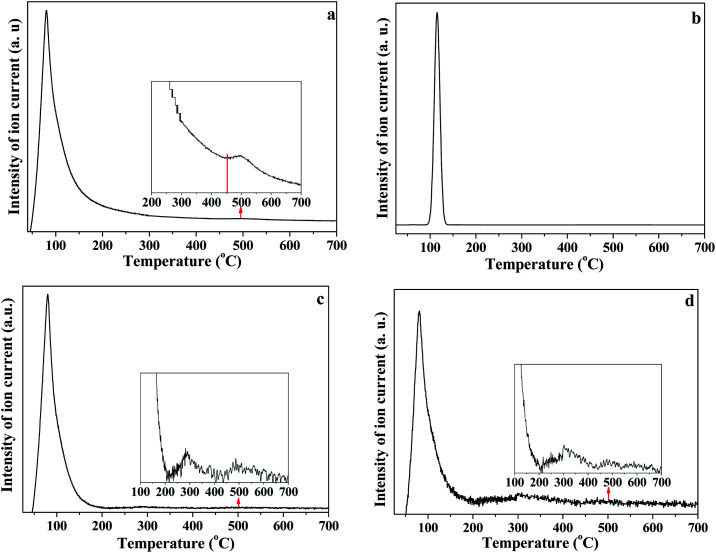
(a) HOD, (b) D_2_O, (c) OD and (d) D release curves during heating of K-D_2_O (a) *m*/*z* = 19; (b) *m*/*z* = 20; (c) *m*/*z* = 18; (d) *m*/*z* = 2.

As illustrated in Fig. 8, the release of HOD (*m*/*z* = 19), D_2_O (*m*/*z* = 20), OD/H_2_O (*m*/*z* = 18) and D (*m*/*z* = 2) is shown in panels (a), (b), (c) and (d), respectively. The first weight loss stage (30–150 °C) corresponded to the release of free re-adsorption D_2_O (Fig. 8b). The HOD (Fig. 8a) could be derived from the hydrogen–deuterium exchange in water vapor,^46^ because the D_2_O reagent may contain trace H_2_O. The release of D (Fig. 8d) can be assigned as the fragment ion from D_2_O. The results shown in Fig. 8c could represent the trace H_2_O content in D_2_O reagent, or the ion of OD resulting from the ion fragment from D_2_O. In the temperature range of 200–450 °C, HOD, OD/H_2_O and D could be detected. These substances correspond to the release of intercalated water in kaolinite. The release of HOD and D indicated that the re-adsorption D_2_O entered the intercalation of kaolinite and hydrogen bonded with the original interlayer water. When the temperature was higher than 400 °C, HOD, OD/H_2_O and D still could be detected, even when the temperature reached 650 °C. The results confirm the inferences from the *in situ* DRIFT spectra, that the majority of adsorption D_2_O could be removed at 650 °C. AS shown in Fig. 8b, the D_2_O molecule was barely detected when the temperature was higher than 150 °C, indicating that the re-adsorption water removed during this stage mainly interacted with kaolinite *via* hydrogen bond.

In order to investigate the changes in crystal structure and chemical composition of kaolinite during the dehydroxylation process, the DK, DK-500, DK-600, and DK-650 samples were analyzed by XRF and XRD.

The XRF results are shown in Table 5. For DK, DK-500 and DK-600 samples, the contents of major components in kaolinite (SiO_2_, Al_2_O_3_) were approximately unchanged. However, for DK-650 sample, the content of Al_2_O_3_ decreased, and that of SiO_2_ increased. The result indicated that the dehydroxylation process was accompanied by the release of the central atoms from the octahedral, *i.e.*, the removal of Al from the octahedral sheets. During the dehydroxylation process, kaolinite lost O–H groups and was transformed to metakaolinite.^23^ Corresponding to the decrease in Al content, the concentration of Si slightly increased. As for the minor ionic impurities in kaolinite samples, the content of K_2_O, Na_2_O, and SO_3_ showed decreasing trend when the heating temperature increased. The results could be due to the evaporation of alkali metals and the decomposition of alunite, which are found in raw kaolinite (see Table 1). As heating temperature increased to 650 °C, the concentration of Fe_2_O_3_ and CaO remained approximately unchanged, and that of TiO_2_ slightly increased. The results demonstrated that the contents of minor ionic impurities (Fe_2_O_3_, CaO and TiO_2_) did not decrease during the dehydroxylation process. The concentration changes of the three minor ionic impurities could also be attributed to the decrease of Al_2_O_3_ content.

**Table 5:** XRF analysis of kaolinites calcined at different temperatures (wt%)

Sample	SiO_2_	Al_2_O_3_	Fe_2_O_3_	TiO_2_	K_2_O	SO_3_	Na_2_O	CaO	Others
DK	49.03	48.41	0.47	0.22	0.47	0.76	0.15	0.06	0.43
DK-500	49.42	48.30	0.49	0.23	0.49	0.72	0.12	0.06	0.17
DK-600	49.24	48.47	0.46	0.22	0.38	0.68	0.11	0.06	0.37
DK-650	50.68	47.17	0.49	0.26	0.36	0.55	0.10	0.06	0.33

**Fig. 9:**
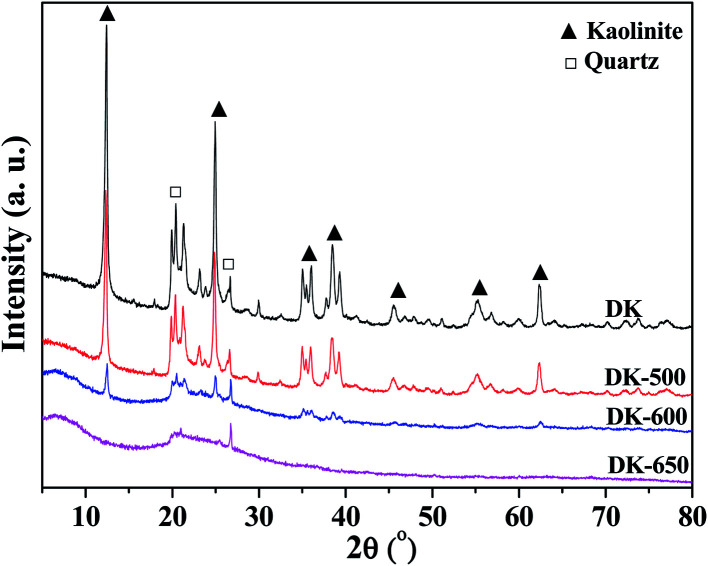
XRD patterns of dried kaolinite and kaolinites calcined at different temperatures.

Fig. 9 shows the XRD results. Two components, kaolinite and quartz, could be obviously found in the XRD patterns of kaolinite samples. The intensities of kaolinite peaks slightly decreased when temperature was higher than 500 °C, indicating that the kaolinite crystal structure initially decomposed at the temperature. When the temperature increased to 600 °C, the XRD pattern showed amorphous structure with partial crystalline peaks of kaolinite and quartz, suggesting the incomplete dehydroxylation and the decomposition of the crystal structure of kaolinite. At 650 °C, the XRD pattern showed amorphous pattern with the trace crystalline peak of quartz, implying the amorphous structure of metakaolin. The diffraction angles for quartz and kaolinite corresponded to those found in the literature .^47,48^

### 3.4 Mechanism of interaction between hydroxyl groups in kaolinite and re-adsorption water

**Fig. 10:**
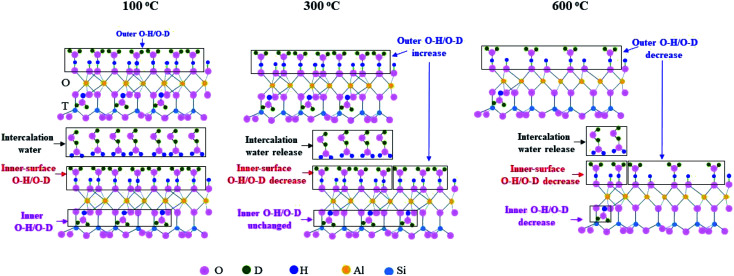
Changes of O–H groups and O–D groups of K-D_2_O at 100 °C, 300 °C and 600 °C.

Based on the above discussion and the kaolinite structure reported in references,^10,49,50^ the changes of O–H groups and O–D groups in K-D_2_O at 100 °C, 300 °C and 600 °C are summarized in Fig. 10. When the temperature increased from 100 °C to 300 °C, two layers of kaolinite slipped away from each other, promoting the increase of outer O–H/O–D groups, the decrease of inner-surface O–H/O–D groups, and the release of intercalation water. At 600 °C, the contents of outer O–H/O–D groups, inner-surface O–H/O–D groups, and inner O–H/O–D groups would decrease dramatically because of dehydroxylation of kaolinite.

### 3.5 Significance of the results to the production of shale gas

Our results proved that all four types of O–H groups in kaolinite would affect the desorption behaviors of methane when water existed in kaolinite. The inner-surface O–H groups would have more obvious effect on the desorption behaviors of methane than the other types of O–H groups. The inner-surface O–H groups would be transferred into outer O–H groups between 100 °C and 300 °C. Thus, the temperature range of 100–300 °C was suggested for heat treatment of the kaolinite to decrease the content of inner-surface O–H groups, thereby reducing the amount of re-adsorption water. Moreover, if the kaolinite could be heated at 650 °C, the effect of re-adsorption water on the desorption behaviors of methane would almost be eliminated.

## 4 Conclusions

The DRIFT vibration at 3670 ± 4 cm^−1^ could be due to the outer O–H groups of the octahedral sheet on the upper surface of kaolinite microcrystal structure rather than a type of inner-surface O–H group. All types of O–H groups in kaolinite could be exchanged into O–D groups when soaking the dried kaolinite in D_2_O at room temperature. Most of the re-adsorption D_2_O was initially adsorbed on the inner-surface sites of kaolinite. As heating temperature increased from 100 °C to 300 °C, two layers of kaolinite slipped away from each other, resulting in the transformation of the inner-surface O–H/O–D groups into outer O–H/O–D groups. The inner-surface O–D groups, the outer O–D groups, and O–D group vibration at 2695 ± 3 cm^−1^ were gradually removed at 500 °C, and the inner O–D groups were removed at 600 °C, whereas all the O–D groups were approximately thoroughly removed at the temperature of 650 °C.

## Conflicts of interest

There are no conflicts to declare.

## Supplementary Material

RA-010-D0RA01905D-s001
