# Supplementary material for: In situ study on interactions between hydroxyl groups in kaolinite and re-adsorption water
Source: RSC Adv. 2020 Apr 30;10(29):16949–58. doi: 10.1039/d0ra01905d (PMC9053203; doi:10.1039/d0ra01905d)
Supplement: RA-010-D0RA01905D-s001 [file RA-010-D0RA01905D-s001.pdf]

Electronic Supplementary Material (ESI) for RSC Advances.
This journal is © The Royal Society of Chemistry 2020

Supporting information

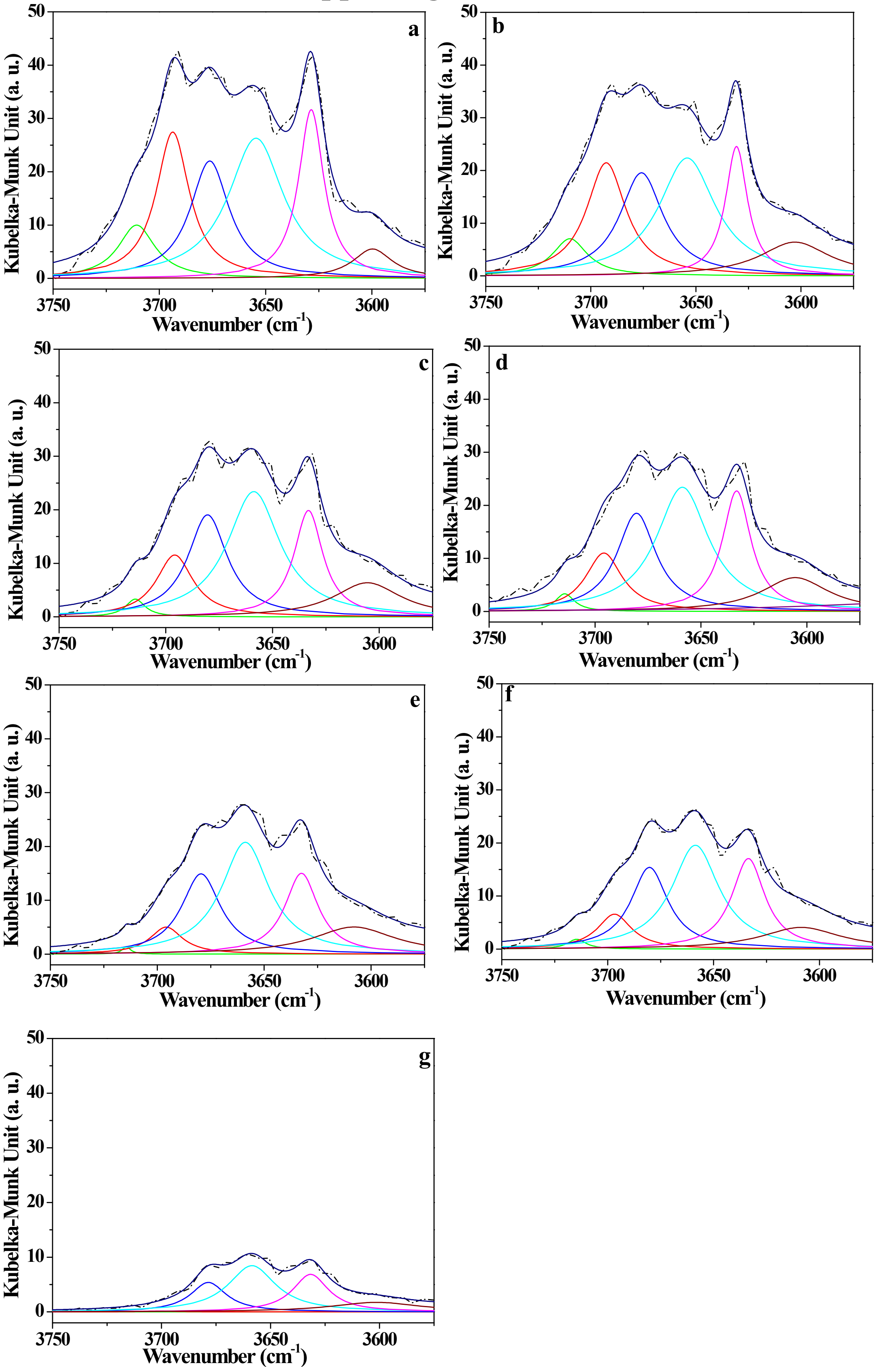

Fig. S_1 O-H region deconvolution of kaolinite containing re-adsorption D_2O heated at 100 ^oC (a), 200 ^oC (b), 300 ^oC (c), 400 ^oC (d), 500 ^oC (e), 600 ^oC (f) and 650 ^oC (g)

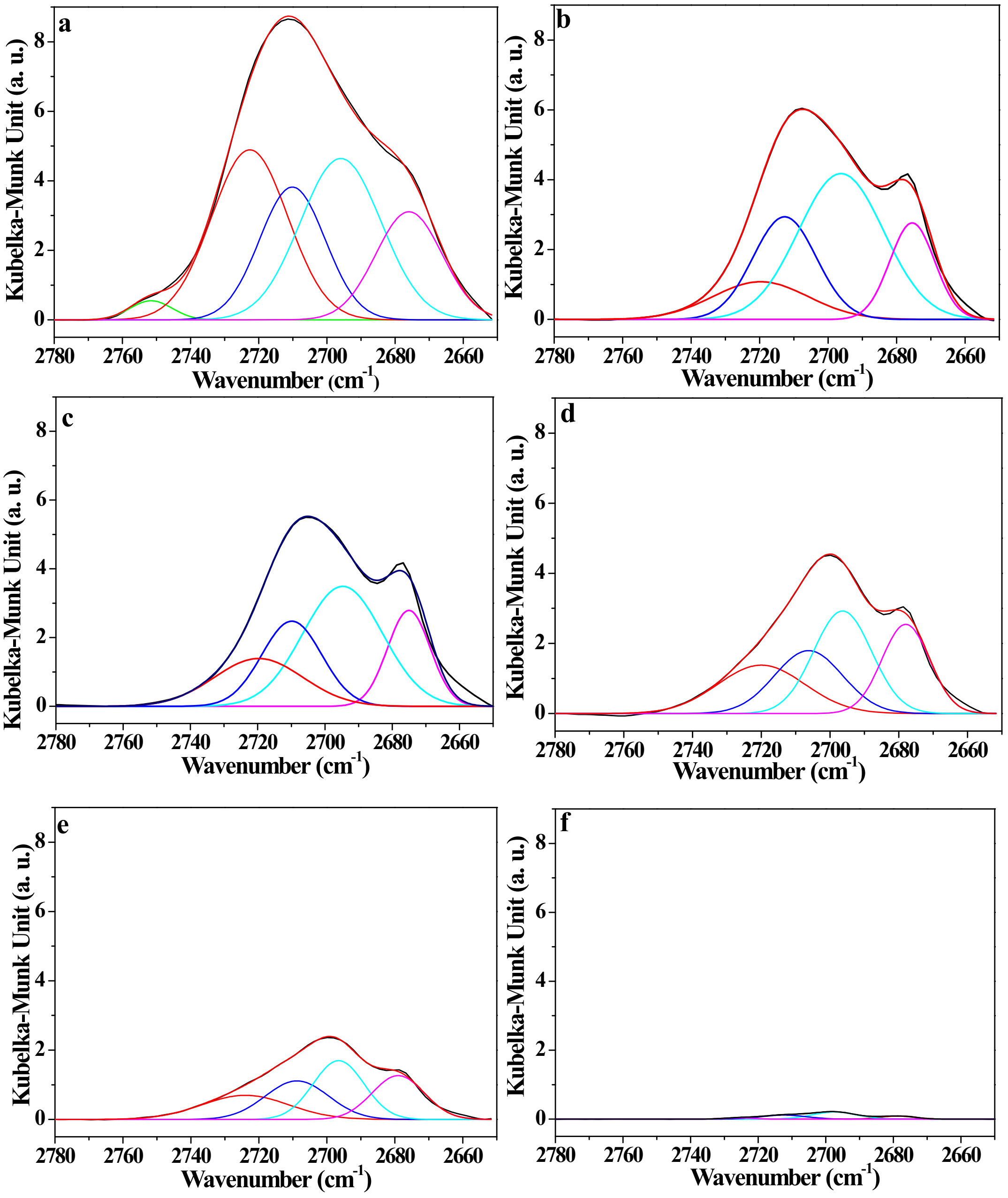

Fig. S_2 O-D region deconvolution of kaolinite containing re-adsorption D_2O heated at 200 °C (a), 300 °C (b), 400 °C (c), 500 °C (d), 600 °C (e) and 650 °C (f)
